# A semi-local neighborhood-based framework for probabilistic cell lineage tracing

**DOI:** 10.1186/1471-2105-15-217

**Published:** 2014-06-25

**Authors:** Anthony Santella, Zhuo Du, Zhirong Bao

**Affiliations:** 1 Developmental Biology, Sloan-Kettering Institute, 1275 York Avenue, New York, New York 10065, USA

**Keywords:** Image processing, Microscopy, In vivo, Cell tracking, Developmental biology

## Abstract

**Background:**

Advances in fluorescence labeling and imaging have made it possible to acquire *in vivo* records of complex biological processes. Analysis has lagged behind acquisition in part because of the difficulty and computational expense of accurate cell tracking. *In vivo* analysis requires, at minimum, tracking hundreds of cells over hundreds of time points in complex three dimensional environments. We address this challenge with a computational framework capable of efficiently and accurately tracing entire cell lineages.

**Results:**

The bulk of the tracking problem—tracking cells during interphase—is straightforward and can be executed with simple and fast methods. Difficult cases originate from detection errors and relatively rare large motions. Therefore, our method focuses computational effort on difficult cases identified by local increases in cell number. We force these cases into tentative cell track bifurcations, which define natural semi-local neighborhoods that permit Bayesian judgment about the underlying cell behavior. The bifurcation judgment process not only correctly tracks through cell divisions and large movements, but also offers corrections to detection errors. We demonstrate that this method enables large scale analysis of *Caenorhabditis elegans* development, an ideal validation platform because of an invariant cell lineage.

**Conclusion:**

The high accuracy achieved by our method suggests that a bifurcation-based semi-local neighborhood provides sufficient information to recognize dependencies between nearby tracking choices, and to interpret difficult tracking cases without reverting to global optimization. Our method makes large amounts of lineage data accessible and opens the door to new types of statistical analysis of complex in vivo processes.

## Background

Advances in fluorescence labeling and imaging have made it possible to acquire *in vivo* records of complex biological processes at unxcbuprecedented spatial and temporal resolution. The largest and most striking datasets are produced by *in toto* imaging of embryonic development based on fluorescent nuclear labels [1-7]. The true value of *in toto* imaging lies in the possibility of tracing the entire cell lineage that underlies development. However, this has not yet been possible in organisms larger than the nematode *C. elegans*.

A major barrier to cell lineage tracing is the technical accuracy of image analysis. To produce even a moderately intact cell lineage high accuracy is needed to track hundreds of cells over many time points and through multiple rounds of cell division. Correct lineage fragment size is critical as it constrains subsequent biological analysis. Accuracy is difficult to attain because cells are densely packed, and this is exacerbated by limited optical resolution due to sample properties and the need to minimize photobleaching and phototoxicity.

The challenging nature of the image analysis task is further compounded by the massive size of *in toto* imaging datasets, often reaching multiple terabytes for the 4D imaging of whole organisms. A practical solution requires a careful balance between algorithmic complexity and computational efficiency.

A number of qualitatively different approaches have been applied to cell tracking. The most common class of approach separates detection and tracking into independent steps for the sake of efficiency and modularity. A popular strategy to match detected cells over time is constrained linear optimization, which assumes independence between matches that are not mutually exclusive [8]. Matches can be made between detections at successive time points, [9,10] or between tracklets built by other methods [11,12]. This approach is inherently local to a match and its alternatives, making it difficult to incorporate semi-local dependencies that are important for resolving ambiguities due to detection errors. For example, detection false negatives (FN) have been handled by supplementing 1:1 and 1:2 division matches with 2:1 and 1:2 apparent merge and split events [13]; however, the merge and presumed subsequent split events remain independent. Global optimization, even under the assumption of independence between non-exclusive cases, is computationally intensive particularly when processing large data sets with standard hardware. More complex optimization formulations can account for scoring dependencies among non-exclusive matches [14] at the cost of additional computation and approximate rather than exact solutions.

A second general class of methods simultaneously detects and tracks objects. Methods for detecting spatiotemporal objects at low cell density [15,16] provide regularized segmentation, but cannot model the ambiguities of motion in crowded images. Tracklets created with this approach have been supplemented with the linear matching methods described above, but the method remains constrained by the limits of each step [17]. Particle filters [18,19] provide a valuable statistical framework for maintaining a multi-modal picture of movement ambiguity given motion and appearance models for an object, however, this rigorous statistical footing tends to be abandoned when dealing with changes in number of tracked objects, a key source of ambiguity in tracking complex tissues. Randomized or Monte-Carlo optimization methods can be used to evolve tracking results under arbitrarily complex scoring schemes [20], though typically at high computational cost. Given these challenges, in practice optical flow methods [21] are often used to characterize motion patterns without attempting to track individual objects.

**Figure 1:**
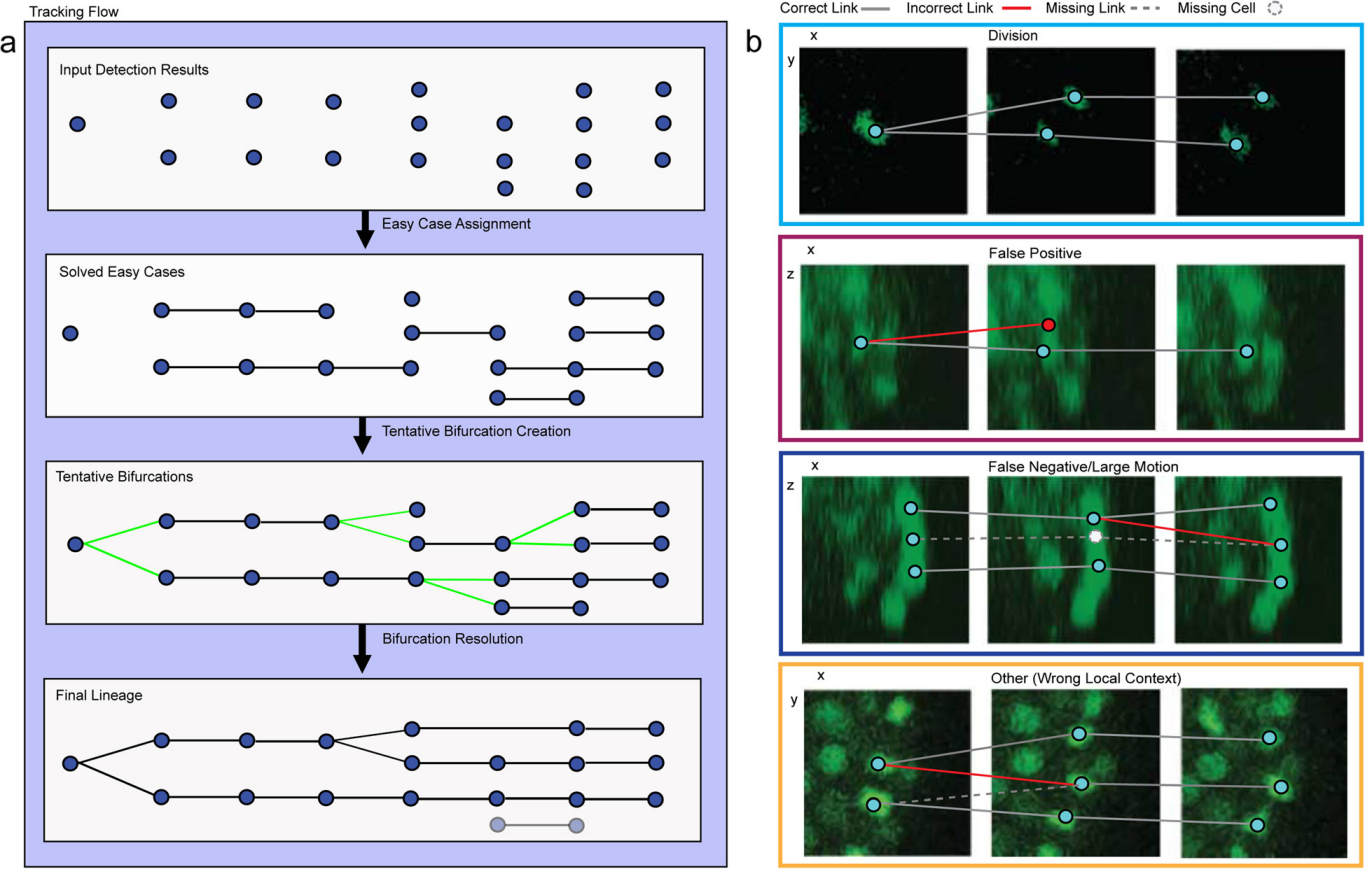
**Approach overview and bifurcation origins. a**. Diagram of the steps in the lineage construction process. **b**. Typical example of each of the four classes of tentative bifurcation, showing the correct (union of correct and missing links) and tentative bifurcation (union of correct and incorrect links) lineage superimposed on image data.

We present a layered greedy approach to achieve high tracking accuracy with computational efficiency (Figure 1a). We perform detection and tracking in separate steps. In the vast majority of cases simple methods based on nearest neighbor (NN) matching are sufficient to track a cell to the next time point. This nearest neighbor assumption is violated only in a small, but significant, fraction of cases such as sudden movements during cell division. We focus on the specific problem of highly accurate tracking under imaging conditions that largely satisfy this limited motion constraint (e.g. no large global motion). Our goal is high accuracy without the cost of global matching methods. Instead, we devote computational effort to solving local ambiguities and the complications caused by detection errors (Figure 1b). We observe that these difficult cases are unified by a local change in the number of nuclei present. As such, they can be detected and solved by forcing tentative bifurcations into existence wherever the local number of nuclei increases. Along with corresponding points of decreasing local cell count, these tentative bifurcations define semi-local neighborhoods where all possible interpretations can be consistently scored within a unified Bayesian framework. Based on classification of bifurcation origin, correct matches are made and detection errors are corrected. Finally, we demonstrate the performance of this approach by tracing the embryonic cell lineage of *C. elegans* as the known invariant cell lineage provides an unambiguous ground truth.

## Methods

In the first step of our layered approach we exploit the minimal movement assumption permitted by frequent imaging. We use *c*_*i,t*_ to denote an individual identified nuclear object at time t. When *c*_*i,t*_ is tracked onto *c*_*i,t+1*_ we refer to the two as linked. Let *NN(c*_*i, t1*_*, t2)* denote the nucleus at time point t2 that is the closest to *c*_*i,t1*_ (excluding *c*_*i*_ itself if *t1 = t2*). Under ideal imaging frequency, the displacement of any given nucleus *c*_*i,*_ from t to t + 1 should satisfy

(1) $$\mathrm{distance}\left(c_{i,\;t},\;\mathrm{NN}\left(c_{i,\;t},\;t+1\right)\right)\;<\;\mathrm{distance}\left(c_{i,\;t},\;\mathrm{NN}\left(c_{i,\;t},\;t\right)\right)/2$$

In a typical *C. elegans* dataset, 99% of the movements per time point satisfy this condition (Table 1). Faster motion does not mean the NN is necessarily a wrong match but suggest it may be unreliable.

**Table 1:** 1:1 links

	**Correct 1:1 links**	**Safe subset of NN links**		**Safe, mutual, non-conflicting NN links**	
		**Correct**	**Incorrect**	**Correct**	**Incorrect**
Detection ground truth	140235	139126	53	137163	5
Segmented results	139151	138027	434	132878	219

Column 1 gives the number of 1:1 links in the detection ground truth and in the computed segmentation result. Detection FN make up the difference between the numbers. Column 2: Safe Subset of NN gives the number of potential NN links forward from ground truth or detected cells that pass the safe distance test (equation 1). Links that pass are subdivided into correct and incorrect categories. Column 3 gives the number of potential NN links that pass all 3 tests (equations 1, 2, 3) also broken down into correct and incorrect links.

In expectation of the complications caused by occasional large movements and detection errors, we take an initially conservative approach and only aim for solutions to unambiguous 1-to-1 mappings. Therefore, in addition to equation (1), we further require two nuclei to be mutual NN:

(2) $$\mathrm{NN}\left(c_{i,\;t},\;t+1\right)\;=\;c_{j,\;t+1}$$

*NN*(*c*_*j*, *t* + 1_, *t*) = *c*_*i*, *t*_

And non-conflicting matches:

(3) $$\mathrm{for}\;\mathrm{all}\;k\;\mathrm{at}\;t+1\;\mathrm{NN}\left(c_{k,\;t+1},\;t\right)=c_{i,t}\;\mathrm{iff}\;k=j$$

*for all k at t NN*(*c*_*k*, *t*_, *t* + 1) = *c*_*j*,*t* + 1_ *iff k* = *i*

In our test set of *C. elegans* data, these three conditions cover 95% of the 1-to-1 links with only .16% error (Table 1). These links represent a largely fixed foundation for further tracking, though as explained below they can be modified under certain circumstances.

### Construction of tentative bifurcations and their neighborhoods

**Figure 2:**
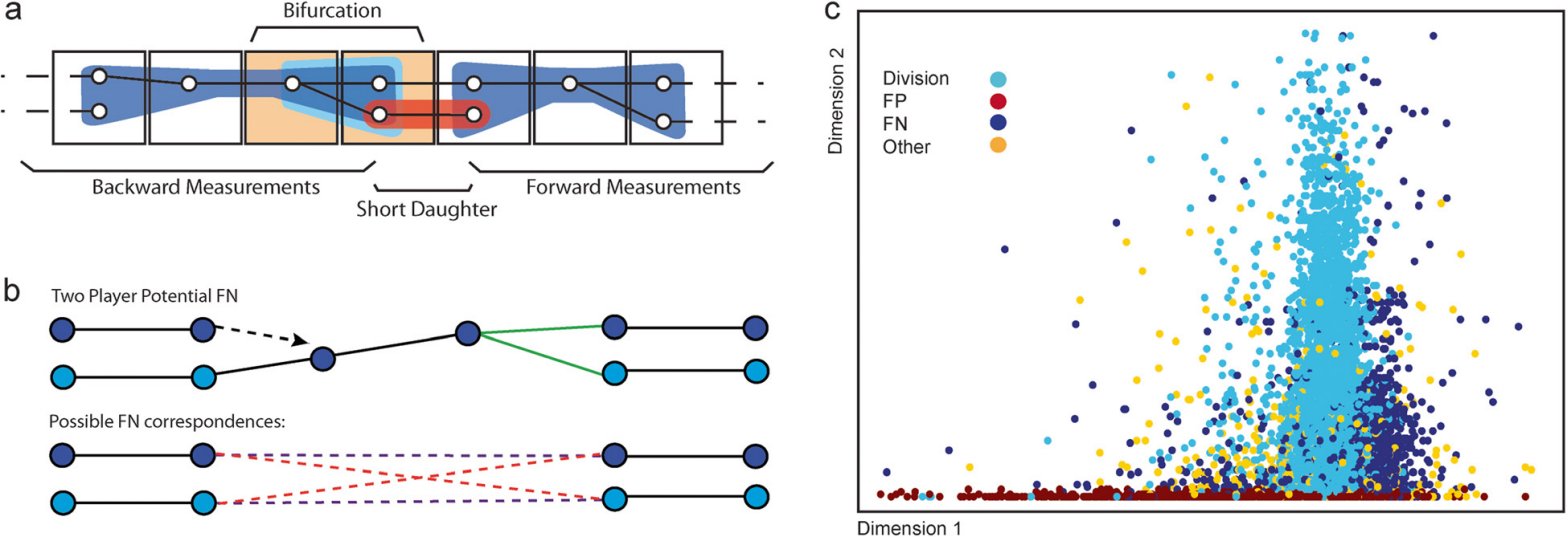
**Bifurcation judgment and false negative resolution. a**. A schematic overview of the semi-local neighborhood used to measure tentative bifurcations and choose their resolution. Sub-regions of the neighborhood that are particularly relevant to specific scenarios are colored accordingly. **b**. Resolution of a typical FN showing how the matching of the ends of a potential FN is performed. The top panel shows the links between detections of two cells, defining a semi-local neighborhood with a bifurcation in green and a NN claim that does not correspond to a link dotted. The lower panel illustrates how matching is evaluated at the putatively correctly segmented endpoints, ignoring links and detection events during the proposed FN (which if there is a FN are unreliable). Each possible match is represented by a different colored pair of dotted lines. **c**. Illustration of two dimensions of the high dimensional measurement vector used to distinguish different bifurcation causes. It illustrates the relatively consistent appearance of many bifurcations with a particular origin, but also that appearances vary widely in a minority of challenging cases.

The remaining cases are triggered by cell divisions, cell movements outside the bounds established in eq (1, 2 and 3), and nuclear detection errors. An increase in cell number within the local neighborhood coincides with an error, or follows the error in the case of FN (Figure 1b). A local decrease in cell number also precedes large cell movements and detection FN that terminate a track. We handle the local cell number increase by forcing bifurcations, that is, 1-to-2 matches from t to t + 1. The two bifurcating tracks are paired with a terminating track in an earlier spatiotemporal window when possible. Together, these define a semi-local neighborhood that provides valuable information to diagnose and correctly track the underlying motion (Figure 2a).

For each cell that does not have a match at the previous time point after the initial 1-to-1 tracking, we identify an optimal bifurcation. Possible bifurcations are pruned by a distance cutoff (Additional file 1: Bifurcation Construction), and evaluated based on morphology and motion to maximize the liklihood of correctly identifying real cell divisions. Divisions can be recognized by their characteristic progression in cell appearance. These include the formation of the metaphase plate, symmetric condensed appearance, as well as motion and relative position of the nascent nuclei (see Additional file 1: Feature Details). These features are combined into a feature vector to score the likelihood of a bifurcation being a true cell division.

We match bifurcations to preceding terminating tracks in the nearby spatiotemporal window by selecting the option that minimizes the total displacement of all relevant nuclei. A typical example of how a simple pairing of a terminating track and a bifurcation is evaluated for minimal movement is shown in Figure 2b. Additional situations are provided in Additional file 1: Neighborhood Construction.

### Classification

The semi-local neighborhood constructed above provides a natural framework to solve situations that challenge the naïve nearest neighbor method. Our approach defines a minimal spatiotemporal neighborhood that contains much of the information used by human experts to solve such cases. It offers a balance between computational complexity and accuracy. Specifically, these neighborhoods contain the defining features to distinguish all situations in the four classes of bifurcations given below (Figure 1b). Particular areas of the neighborhood (see colored regions in Figure 2a) often dramatically signal a specific underlying cause. Our approach simultaneously weighs subtle cues from the entire neighborhood in order to resolve ambiguous cases, much like a human does. We outline these features here and provide the technical details in Additional file 1: Feature Details.

(1) Cell divisions: The bifurcation is sufficient to capture the characteristic sequence of cell morphologies and the mitotic movements.

(2) Pairs of two non-dividing nuclei: These are caused by excessive movement of one of the two nuclei, or a FN in nuclear detection at the previous time point. In this case the morphology of the three detection events involved in the bifurcation resembles that of interphase nuclei. Excessive movement or detection FN also generate a dangling end (1-to-0 match) within a nearby spatiotemporal window that can be matched in terms of morphology and position.

(3) Detection false positives (FP): Because of their typical origin in optical distortion and fluorescence heterogeneity within nuclei, FPs in nuclear detection display distinct morphology and relative positions to nearby nuclei [22]. In addition, because of the low FP rates, tracks consisting of FP detections typically only last for a few time points marking them as likely candidates for deletion.

(4) Other cases: This class is treated as an umbrella class for examples that do not belong to the three classes outlined above. Cases in this class lack a unifying set of intrinsic features. This is because the local neighborhood is insufficient to explain and resolve the bifurcation, such as a nascent nucleus being linked to a wrong mother.

We use Naïve Bayesian inference for probabilistic classification of the bifurcations. We measure all relevant features described above to create a combined feature vector *M = [m*_*1*_*…m*_*n*_*]* (Figure 2c). We then calculate *P(class*_*i*_*|M) P(class*_*i*_*)P(M|class*_*i*_*).* For simplicity, we assume independence of the features. Priors and feature distributions are fit from training data. In our *C. elegans* dataset, the temporal resolution of imaging and the detection accuracy drive ambiguity down to a level where the prior frequency of cell division is comparable to the other classes combined (Table 2).

**Table 2:** Division statistics

	**Correct**	**Incorrect**
Real divisions in data	2060	-
Initial Bifurcations	1881	1594
Final Divisions	1855	87

Correct and incorrect divisions in the data set at each step of processing. Incorrect divisions are bifurcations that originate from detection errors or fast motion.

### Tracking actions

Once a neighborhood is assigned to one of the four classes, we perform the following actions in terms of cell tracking:

(1) Cell divisions: The bifurcation is accepted.

(2) Pairs of two non-dividing nuclei: During the construction of a neighborhood, a set of 1-to-1 matches between the involved nuclei has been computed to minimize the overall movement. These matches are accepted as the optimal tracking under the 1-to-1 hypothesis. In the case of FN in detection, the dangling end is more than one time point away from the bifurcation. The gap in nuclear detection is filled by linear interpolation.

(3) Detection false positives. If a bifurcation falls in this class, the shorter track is deemed an FP and deleted.

(4) Other. In this class the bifurcation is not the appropriate local neighborhood to resolve the situation. The appearance and motion pattern of non-dividing cells provides the information to infer which of the two “daughters” is a better match (see Additional file 1: eq 1). The worse match is then attached to the next best bifurcation (see above), and the bifurcation classification is iterated. If all acceptable bifurcations are exhausted, the cell is left without a backward link, that is, as an unexplained cell appearance.

## Results and discussion

Performance of the tracking algorithm was evaluated on *C. elegans* embryos imaged from the 4-cell to 350-cell stage, which covers ~4 hours of embryogenesis with ~200 time points at 75 second intervals, totaling approximately 25,000 nuclear objects to be tracked per embryo. Bifurcation probability models and other parameters were trained on 10 embryos where the lineages were manually examined and corrected. Performance was evaluated on a set of 5 independent embryos (some key frames of example image data are provided as Additional file 2). Global instantaneous accuracy (one minus the total number of instantaneous errors in all frames divided by the number of cells in all frames) was measured for each embryo at the end of all tracking steps.

**Figure 3:**
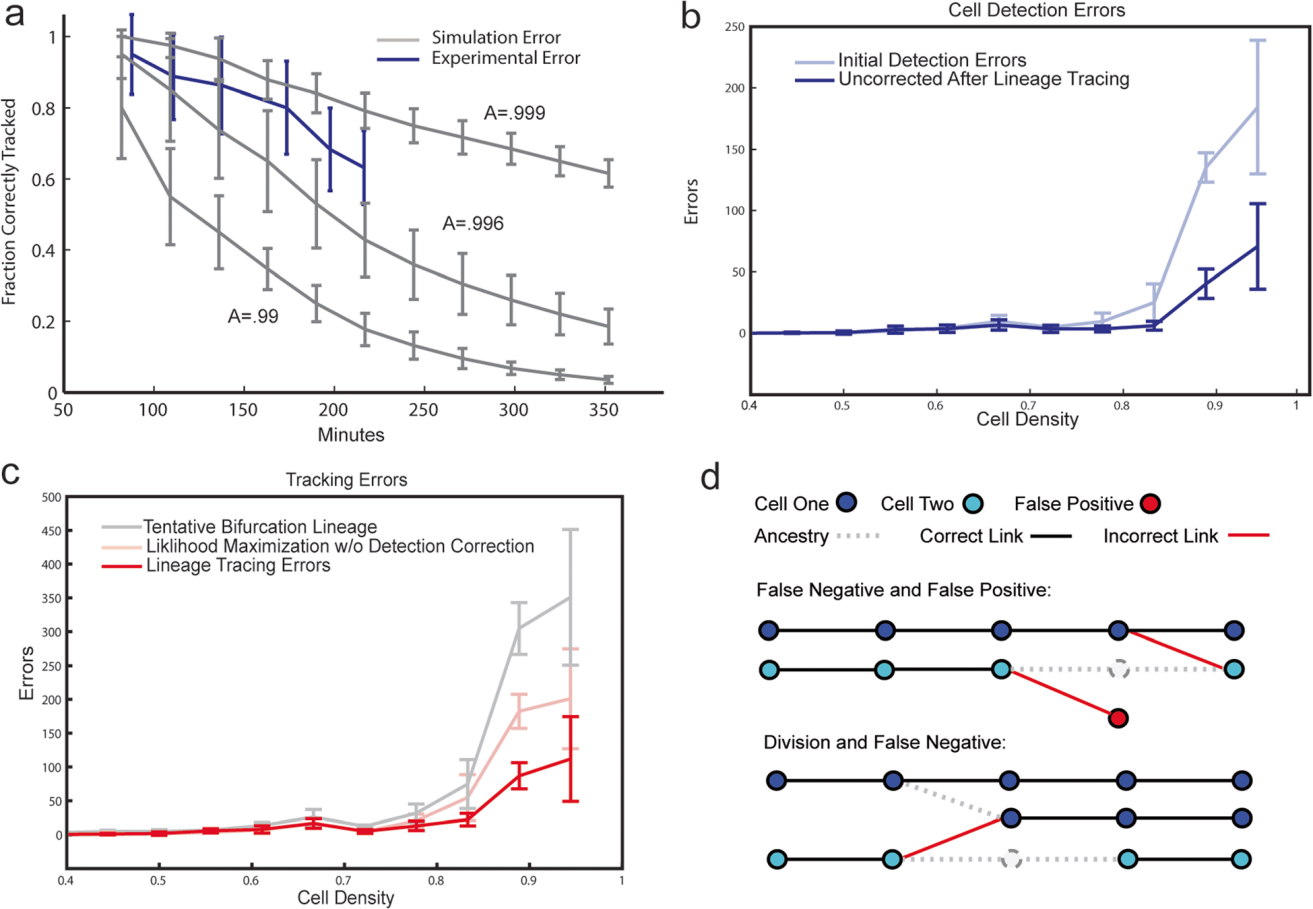
**Tracking performance. a**. Cumulative error accumulation along tracks over time. Mean and variation is shown over embryos in cumulative accuracy, specifically the fraction of AB (from AB8 to AB256) cells correctly tracked. Experimental results are in blue and show the fraction of cells tracked correctly from the beginning through each generation. Each gray line is the result of simulation of cumulative error over the same time period. A uniform average error rate and average cell cycle length is assumed. Errors are introduced at each cell and frame in the simulated lineage at the instantaneous error rate, and a cell is considered wrong if any errors have been introduced during its history. **b**. Initial cell detection error counts (FN + FP), and counts after bifurcation resolution as a function of cell density. Density is measured as the inverse of space between nuclei. For cell *c*_*i,*_*density = (radius(c*_*i*_*) + radius(NN(c*_*i*_*,t))/distance(c*_*i,*_*,NN(c*_*i*_*,t))*. A density of one means no gap between the nuclei is present. **c**. Tracking error count (FN + FP) in the tentative bifurcation set and in the final results of our method. This is contrasted with tracking errors present in a more naive link likelihood maximization scheme. As above both error counts are displayed as a function of cell density. **d**. Difficult cases. Multiple detection errors in the same area presents difficulty for our method. In the first case the confluence of a FN and FP make it impossible to generate the exact correct answer. In the second example a division and FN together violate our methods assumption that both ends of a FN will be marked by a change in network topology, making the correct FN resolution impossible.

Our greedy approach effectively decomposes the combinatorial global optimization problem into appropriate local problems, as demonstrated by the accuracy of the result and its computational efficiency. The overall instantaneous accuracy through 350 cells is 99.6 + −.1% (variance calculated on a per embryo basis). In terms of cumulative track accuracy ~60% of the cells in the last frame are correctly tracked from the beginning through ~200 time points and 6 rounds of cell divisions with no errors intervening along the way (Figure 3a). Tracking through to 350 cells takes approximately 1.5 seconds/volume with an additional 6 seconds/volume required for segmentation using a single threaded implementation of our method on a 2.8 GHz PC with 12GB of RAM.

Accuracy of tracking is inversely correlated with cell density, which also influences detection accuracy (Figure 3b). At lower cell densities (early developmental stages), our method achieves essentially 100% accuracy. When the separation between neighboring nuclei, as measured by the distance between the shell of estimated nuclear boundaries between NNs, drops to ~1 pixel in the test images (or 5% of the distance between detected nuclear centers) the accuracy of tracking drops to 99.4%. Our approach corrects almost all errors in nuclear detection at low cell densities and around two-thirds of errors at high density (Figure 3c).

We further investigated the specific gain of using the semi-local neighborhood to model and correct errors. To this end, we compared our method to the more naive approach of only modeling cell divisions. As shown in Figures 3c, creating cell divisions using the same measurements and picking an optimal likelihood threshold for bifurcation creation produces twice as many errors compared to the full approach. We also compared the performance to our previous approach, which, like many other algorithms used in the field, only generally targets the two obvious situations of cell movement and division [3]. The error rate for the previous tracking method is four times higher based on the same detection results (1.6% + −.4% vs 0.4% + −.1%). In addition to correcting detection errors, the method presented here greatly improves the positive predictive value (PPV) of a division from an average 72.3 to 95.5, while simultaneously increasing sensitivity. This is important because false divisions are particularly troublesome as they qualitatively distort the apparent developmental history and cell cycle length.

Our approach also achieves high accuracy compared to the accuracy of other methods on the same or similar data sets. Reported literature accuracies on the early, less challenging stage of *C. elegans* development up to 180 cells ranges from 97% for a semi-automated approach [23] through 98.9% with a graph cut minimization [15]. A parallel effort [24] used Support Vector Machines (SVMs) to judge the correctness of the bifurcations generated by our previous tracking method [3] however, it only makes a binary judgment of bifurcation correctness and therefore cannot correct detection errors. Other efforts to address late *C. elegans* development have not reported late stage error rates [23] or have achieved similar results at exponentially larger computational cost [20].

Our approach is somewhat dependent on low error rates in detection. It makes the assumption that detection errors reveal themselves by a non-1:1 mapping in the semi-local neighborhood. Compound errors, such as complementary FN and FP (Figure 3d) can violate this assumption making accurate resolution difficult or impossible. Chances of becoming trapped in such situations can be dependent on the exact method and order in which tentative bifurcations are created. The prevalence of ‘Other’ cases in the confusion matrix (Tables 3 and 4) gives some sense of the number of cases that cannot be resolved. Note, however, that this total is exaggerated as the table sums over all tentative bifurcations seen during lineageing, and each unresolvable bifurcation will yield several ‘Other’ bifurcations as different branch points are exhaustively tried. More sophisticated methods for bifurcation resolution, such as creating and resolving tentative bifurcations in order of certainty of classification are one open research area.

**Table 3:** Confusion matrix of classifier on all tentative bifurcations generated while processing training data

	**Other**	**Division**	**FN**	**FP**
Other	935	69	66	85
Division	99	3395	31	68
FN/1:1	79	39	546	43
FP	17	7	28	1416

Rows are correct labels columns are predicted labels.

**Table 4:** Confusion matrix of classifier on test data

	**Other**	**Division**	**FN**	**FP**
Other	636	39	5	35
Division	15	1855	1	10
FN/1:1	116	29	365	14
FP	25	19	13	972

Rows are correct labels columns are predicted labels. This includes both initial bifurcations (Table 2 row 2) and additional bifurcations generated in re-processing of Other cases.

Methods for assigning bifurcation classifications remain to be optimized. We have found our relatively simple and generic Naïve Bayes classifier performs acceptably; achieving ~90% accuracy in identifying bifurcation causes in test data (Tables 3 and 4). Other classifier frameworks, such as SVMs, though not probabilistic, perform strongly in most situations [25] and might be able to solve more difficult cases. More sophisticated probability models could also yield better prediction or better generalization in particular applications. SVMs (or other learning approaches) are not however a panacea. SVMs have been applied to distinguishing true and FP divisions using features qualitatively similar to our bifurcation measurements [24]. Resulting classification accuracy was 88%, lower than our global classification accuracy and significantly lower than our accuracy on this reduced problem (Table 4). This is not a weakness of SVMs but a reflection of the importance of measuring the entire semi-local neighborhood and explicitly modeling the major classes of alternative causes for bifurcations.

Matlab source code distributed under GNU GPL and a compiled Windows binary are freely available: http://sourceforge.net/projects/starrynite/files/starryniteII/.

## Conclusion

The high accuracy of our layered greedy tracking approach suggests that sufficient information is present within a semi-local neighborhood to recognize dependencies between tracking cues, and to interpret difficult cases without reverting to global optimization. This kind of semi-local greedy solution is vital in large scale analysis, such as in toto imaging of complex organisms. In such data sets, global optimization becomes unfeasible due to high computational and memory demands. Tentative bifurcation resolution provides a logical means for framing this task, allowing us to create a unified, and principled statistical model that is intuitive, low dimensional, and easily trained from only corrected lineage results.

Our tracking approach begins with independent detection results but corrects detection errors through bifurcation-based modeling. As our tests show, this approach maintains the computational efficiency of independent detection and tracking while achieving high accuracy. In contrast to traditional tracking methods with strong priors for the continued existence of a tracked object, our approach creates more fragmented results in the most challenging images but avoids the corresponding risk of hallucinating entire sections of track in the case of cell death or long term disappearance due to imaging limitations.

The ability to accurately follow cells over long periods is sensitive to relatively small changes in error rates. Therefore, while the reduction of instantaneous error from 1.6% to 0.4% may appear modest, it makes a crucial difference in lineage analysis. With even slightly worse error rates, as illustrated by the simulation results in Figure 3a, cumulative accuracy quickly degrades. In *C. elegans* embryogenesis, an instantaneous accuracy of 99.6% is just sufficient to follow over half of cells correctly through ~200 time points and 6 rounds of cell divisions to the 350 cell stage, where gastrulation and organogenesis are complete. We note that a >50% cumulative accuracy can enable fully automated single cell analysis by statistical integration of multiple datasets with tracking errors. In general, statistical analyses are enabled by maximizing the correct lineage fragments that are generated. Our approach therefore provides a qualitatively important accuracy level that opens the door to new approaches that can better utilize increasingly large image datasets.

## Availability of supporting data

The data set(s) supporting the results of this article is(are) included within the article (and its additional file(s)).

## Competing interests

The authors declare that they have no competing interests.

## Authors’ contributions

AS conducted the primary algorithm design implementation and evaluation and drafted the manuscript. ZD advised on microscopy concerns and executed imaging experiments. ZB participated in algorithm and experimental design and revised the manuscript. All authors read and approved the final manuscript.

## Supplementary Material

Additional file 1 Supplemental methods. Click here for file

Additional file 2 Example image data, three images with ground truth segmentation. Click here for file
