# Supplementary material for: A semi-local neighborhood-based framework for probabilistic cell lineage tracing
Source: BMC Bioinformatics. 2014 Jun 25;15:217. doi: 10.1186/1471-2105-15-217 (PMC4085468; doi:10.1186/1471-2105-15-217)
Supplement: Additional file 1 — Supplemental methods. [file 1471-2105-15-217-S1.doc]

**Additonal File 1**

**Supplemental Methods**

**Image acquisition**

C. elegans 3D time lapse images were recorded between the two cell stage and hatching with a spinning disc confocal microscope (Zeiss Axio Observer.Z1 with 491-nm and 561-nm lasers) at a temporal resolution of 75 seconds, an axial resolution of 1um and lateral resolution of .254um. To normalize for loss of fluorescence in deeper focal planes and increase in fluorescence later in development, adjustments were made to laser power (ranging from five to thirty percent) and exposure time (from eighty five ms to 120 ms) according to slice and developmental stages. Only data to the ~ 350 cell stage (~220 minutes) was used in this analysis.

Ten training and five test embryos were collected from various strains sharing the [his-72(promoter)::his-24::mCherry + unc-119(+)]; stIs37 [pie-1 (promoter) :: mCherry::H2B + unc-119(+)] transgenes used for lineaging. All strains were grown at 22°C under standard laboratory conditions.

**Bifurcation Construction**

In order to reduce the number of pairwise combinations to be considered, we take a greedy approach to construct bifurcations. In the first step, additional one to one links are made. This is achieved through one of two alternative methods based on user choice. The first method picks the nearest neighbor at the previous time point not already taken. Claims are satisfied in distance order using a sliding threshold up until a maximal distance threshold: *MD1* of .875 * average nearest neighbor (NN) distance. This cutoff corresponds to the maximal normalized speed expected of a non dividing cell (and the closer of the two daughters at a division, as the distance from the parent is frequently asymmetric). The threshold is a configurable parameter and was picked to empirically minimize error after subsequent steps. The second method uses a statistical one to one link likelihood scoring function. It scores a match *ij* as:

$S(ij)=L(correctlink|m_{ij})$ (1)

Where [m_ij_] is set of 1:1 motion and appearance measurements on the two cells (see detailed list of features below) and *L* is the likelihood based on a probability density computed from a multidimensional Gaussian fitted to measurements from genuine 1:1 links. The choice with maximal *S(ij)* is chosen. While the second method appears more sophisticated, its use in the *C. elegans* datasets results in marginally lower accuracy than simply minimizing distance. This is likely because of the highly similar appearance of nuclei and the high variance in appearance measurements due to image noise. In a tissue with more heterogeneous nuclear appearance this might not be the case.

In the second step, all remaining detection events that lack a backward match are made into tentative bifurcations by matching them to a nucleus at the previous time point that already has a forward match. The goal is maximize the probability of a bifurcation being a true cell division. Therefore, bifurcations are chosen that maximize division likelihood using a Gaussian model of division appearance measurements (detailed below). This is done as long as a candidate exists within a maximal distance cutoff: *MD2* of 1.2* average closest neighbor distance. This cutoff corresponds to the maximal normalized speed expected of the farther daughter in a division. The threshold is a configurable parameter and was picked to empirically minimize error after subsequent steps. All claims are satisfied, lowest (best) scoring first, implemented with a sliding threshold. The cost function used here is also a configurable parameter, with a division distance cost function also provided (distance from parent to daughters midpoint) to allow easy initial lineage creation on novel data sets prior to model creation using edited results.

**Neighborhood Construction**

The motivation for neighborhood construction is to collect non-local information needed to score potential false negative (FN) or large motion configurations (class 2 in the 4-class scoring described in the main text) and weigh these against other possibilities. The task for each bifurcation is to identify terminated tracks/dangling ends in a temporal-spatial window prior to the bifurcation. The window is defined as a cylinder in 4D space, with a distance cutoff *(MD2)* and time cutoff that can be tuned by users (8.75 minutes in reported results).

While figure 2a in the main text explains the simplest situation, complications may arise. First, because positional errors in nuclear detection frequently occur upon FN, the dangling end may not correspond to one of the nuclei in the bifurcation (e.g., a “musical chair” shift during tracking). Therefore, an additional step is used to identify other potential players: for a dangling end, its closet neighbor at the next time point is included (dashed arrows in Sup. Figure1).

The match displacement is measured as the average displacement of the best match for all 2 or 3 players at the hypothesized FN/1:1 start and end points. If there are more than 3 players at either end, or the number of players at each end does not match then the simple daughter to dangling end distance is used.

Second there may be more than one dangling end in the temporal-spatial window. Accordingly, this process is repeated independently for each possible match to both daughters and the minimal displacement option with its corresponding cell matches is presumed to be the FN/1:1 match if ultimately the classifier returns a FN/1:1 decision. Segments of track in between the players are linked as a whole to the pair of matched ends that the in-between track endpoints are closest to overall, with the pair furthest from any in-between section of track being interpolated as the FN.


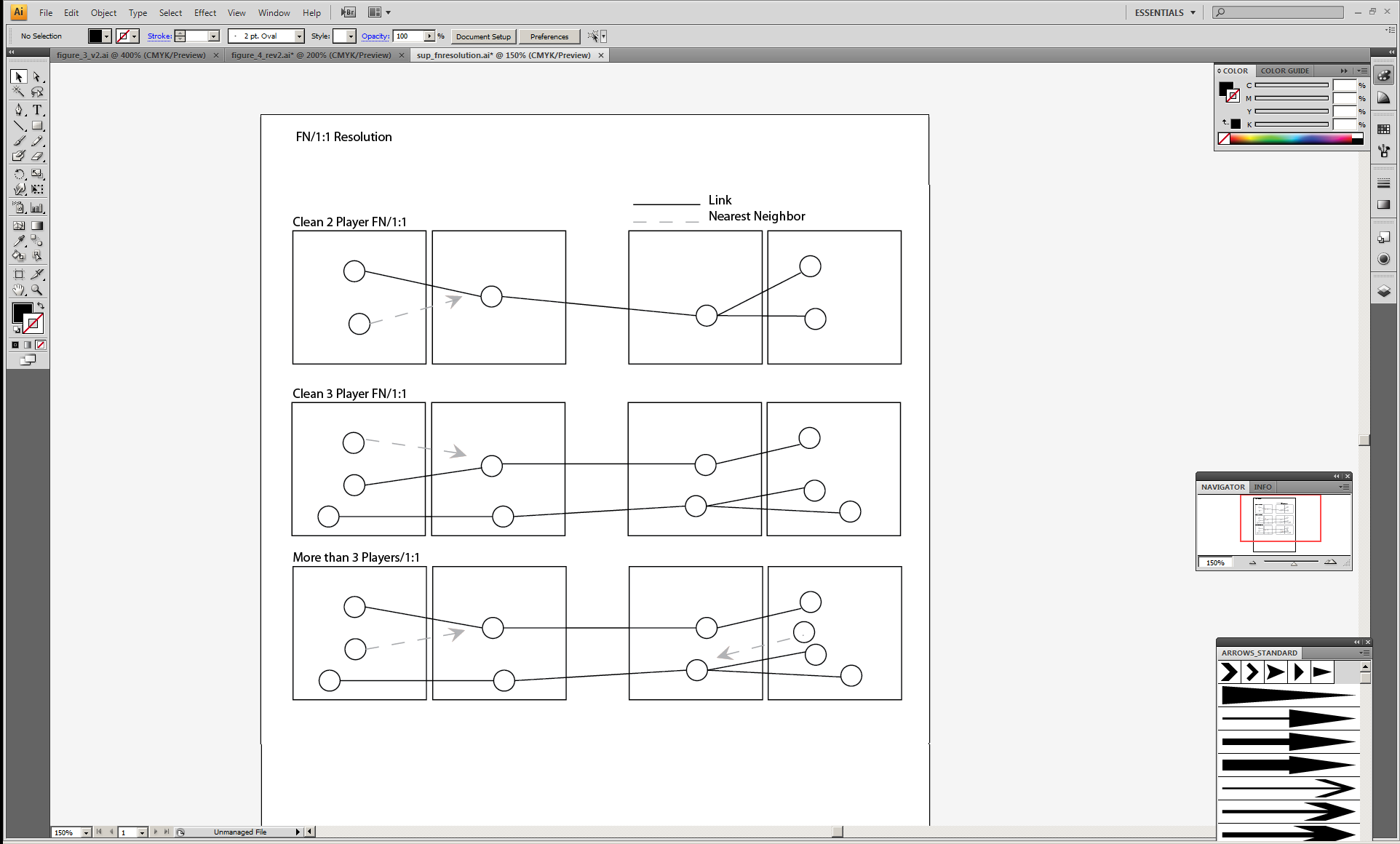


**Supplemental Figure 1** FN/1:1 are scored and resolved based on minimizing the displacement between all conflicting players in 2 and 3 participant cases.

**Bifurcation Classification and Resolution**

Each tentative bifurcation in the data set is examined and processed in temporal order (ordering is arbitrary within single time point) under the assumption that given decreasing image quality throughout development earlier cases are less ambiguous.

The available vectors are concatenated together into a vector M=[m_1_..m_n_] and assigned a maximal a posteriori likelihood explanation using an independent feature (naïve Bayes) probability model:

*Best Explanation = argmax _explanation_ P(explanation)∏_i_ P(m_i_|explanation)*

**Training the Bifurcation Classifier**

The underlying probability model is built from training data by re-processing data sets which have been previously edited to ensure correctness. Training begins by matching the unedited detected cells against the edited lineage. First nuclei at all time points are matched, looking for mutual NN between the data sets within 1 average radius of each other. This separates cells in the edited and unedited lineage into 3 classes, matches, FN and FP. The bifurcation appearance model is trained from labeled bifurcations in the edited results and used to create tentative bifurcations in the unedited data set, training cases for the bifurcation classifier. A tentative bifurcation in the unedited lineage is called a true division if the parent and daughters match to edited cells which are also linked in a division. It is called a FP if at least half of the cells in both daughter stretches during the shorter daughter’s lifetime are labeled as FP. It is called a FN/1:1 if one of the daughters is a descendent of the best scoring FN/1:1 back option. All other situations are given a label of ‘Other’. This creates a training set of tentative bifurcations each with a class label and some set of available measurements of the semi-local neighborhood.

We have experimented with two different underlying classifier structures to characterize this training set. The primary approach (reported performances) creates an independent probability model with priors and likelihood distributions for each ‘case type’ (a combination of available features and outcomes, e.g. gap measurements and the FN/1:1 outcome are unavailable when no corresponding cell decrease is found). Four combinations are possible and given our large corpus of training data there is sufficient data to train this stratified model. A simpler classifier with only one likelihood model per feature and cause which treats ‘case type’ as an additional categorical feature is more appropriate when training data is limited. Given the independent feature model, likelihood terms that cannot be measured for a particular bifurcation are simply left out and the maximum posterior probability is computed over available terms. This approach is more robust when data is limited, (while absent sufficient training data the full model will assign probability zero to previously unencountered events). It does however suffer from slightly degraded classification performance on the full training data (data not shown). This implementation is also provided.

In either scenario the underlying probability density can be modeled as a Gaussian or via kernel density estimation. This also is a user configurable parameter. Kernel density estimation is used in reported results, but a Gaussian model may be more robust with limited training data.

The set of features which is most relevant to decisions might vary from data set to data set. Given our relatively simple Naïve Bayes classifier an additional feature reduction step is used. During model training each of the features in the full measurement vector is tentatively dropped and that feature is removed from use if classification results (on training data) are no better when it is included.

**Feature Measurements:**

Four groups of morphological and motion features are used to characterize the different situations. Their use is summarized in Supplemental Figure 2, and the composition of each group is listed in the four tables below.


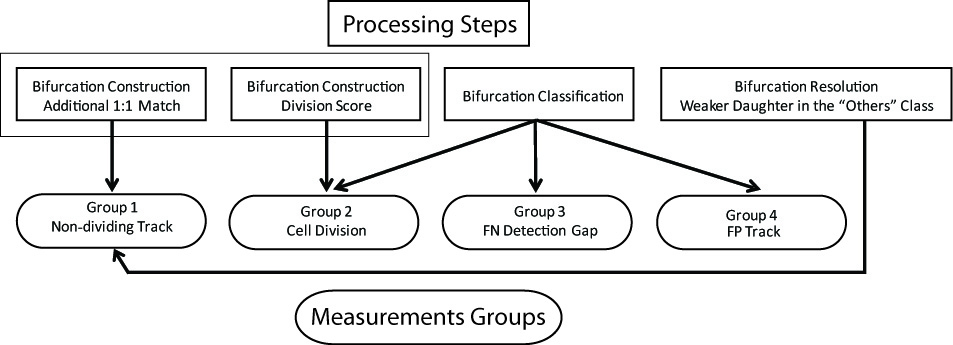


**Supplemental Figure 2** Dependence of processing on Measurement Groups. Steps of local neighborhood construction and resolution are listed in square boxes. Each rounded box represents a corresponding group of feature measurements from the list below. Arrows indicate the dependence of a processes on a group of measurements.

| **Class (underlying cause) of bifurcation** | **Group 2 Cell Division** | **Group 3 FN Detection Gap** | **Group 4 FP Track** |
| --- | --- | --- | --- |
| Cell Division | High | None/Low | Low |
| FN/large motion 1:1 | Low | High | Low |
| FP | Low | None/Low | High |
| Other | Low | None/Low | Low |

**Supplemental Table 1** Relevance of feature groups to identification of particular scenarios.

**Feature Details**

**Group 1: Features for Non-Dividing Cell Tracks**

| Feature | Details |
| --- | --- |
| Cell Lineaging Marker Fluorescence Sum | Sum over voxels within segmented nuclear boundary  sum(cell time t)/sum (cell time t+1) |
| Cell Lineaging Marker Fluorescence Average | Average Sum over voxels within segmented nuclear boundary  average(cell time t)/average( cell time t+1) |
| Diameter change | Maximal diameter of slice in cell segmentation  Diam( time t)/Diam(time t+1) |
| Motion | Magnitude of X,Y component of motion  Magnitude of Z component of motion |

**Group 2: Cell Division Features**

| **Feature** | **Details** |
| --- | --- |
| Parallel drift | Length of projection of parent daughter midpoint distance onto the line between the daughters/ avg NN distance |
| Perpendicular drift | Remainder of displacement between parent and daughter midpoint/avg NN distance |
| X,y extension | X,Y component of distance between daughters/avg NN distance |
| Z extension | Z component of distance between daughters/avg NN distance |
| Cell Lineaging Marker Fluorescence Sum | sum (parent)/sum (daughter1 (nearer)) sum (parent)/sum (daughter2) sum(daughter1)/sum(daughter2) |
| Cell Lineaging Marker Fluorescence Average | average(parent)/average (daughter1 (nearer)) average (parent)/ average sum (daughter2) average (daughter1)/ average (daughter2) |
| Diameter change | diam(parent)/diam(daughter1) diam(parent)/diam(daughter2) diam(daughter1)/diam(daughter2) |
| Parent aspect ratio | Cross sectional aspect ratio. Principle component 1 length/Principle component 2 length computed for 2D point cloud projection onto axial plane of 3D points on segmented nuclear surface |

**Group 3: Features for FN Detection Gaps**

| **Feature** | **Details** |
| --- | --- |
| Gap Length | Length (in minutes) of time gap |
| Cell Lineaging Marker Fluorescence Sum | (cell gap start)/sum ( cell gap end) |
| Cell Lineaging Marker Fluorescence Average | average(cell gap start)/average( cell gap end) |
| Diameter change | Maximal diameter of slice in segmentation before gap/after gap |
| Motion over gap | Average displacement of players over gap(see above)/avg NN distance –used only for gap classification |

**Group 4: Features for FP Tracks**

| **Feature** | **Details** |
| --- | --- |
| Track lifetime | Length (in minutes) of track existence * |
| Cell Lineaging Marker Fluorescence Sum | Sum over voxels within included slice boundary within lineaging normalized by average per nucleus sum within that time point |
| Cell Lineaging Marker Fluorescence Average | Average over voxels within included slice boundary normalized by average per nucleus average within that time point |
| Cell Distance from NN | NN distance within timepoint/avg NN distance at timepoint |
| Cell Aspect ratio | Ratio of height of slices within segmented nucleus/ maximal diameter circular slice approximation |
| Cell Shape model match | Average score of included slices in nucleus segmentation against the slice model used in detection/segmentation |

***** Empirically required to be <=4. That is, if a track is longer than 4, it is not considered as a possible FP track.
