# Supplementary figures and images for: A semi-local neighborhood-based framework for probabilistic cell lineage tracing
Source: BMC Bioinformatics. 2014 Jun 25;15:217. doi: 10.1186/1471-2105-15-217 (PMC4085468; doi:10.1186/1471-2105-15-217)

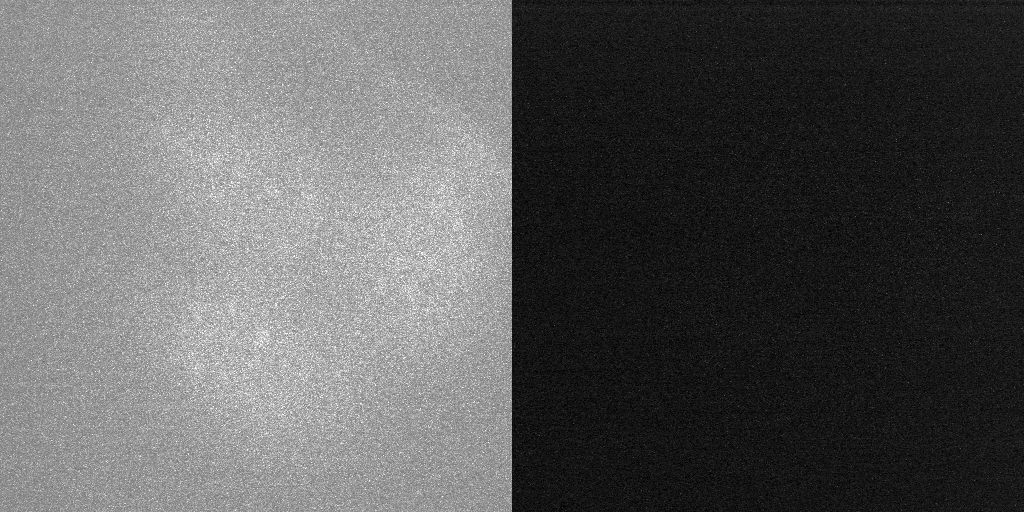

Supplement: Additional file 2 — Example image data, three images with ground truth segmentation. [file 1471-2105-15-217-S2.zip › ZD_RW10425_WT_20100412_2_s1_t101.TIF]

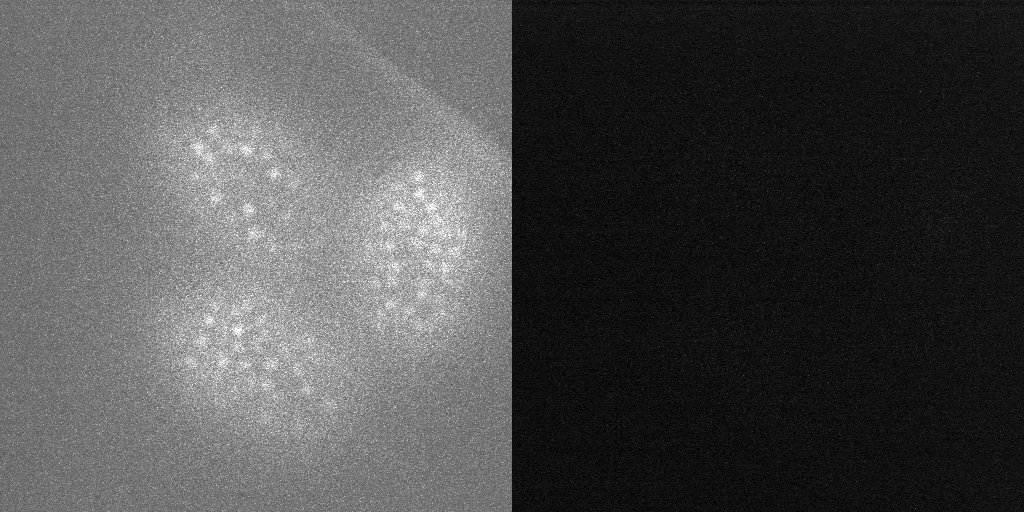

Supplement: Additional file 2 — Example image data, three images with ground truth segmentation. [file 1471-2105-15-217-S2.zip › ZD_RW10425_WT_20100412_2_s1_t151.TIF]

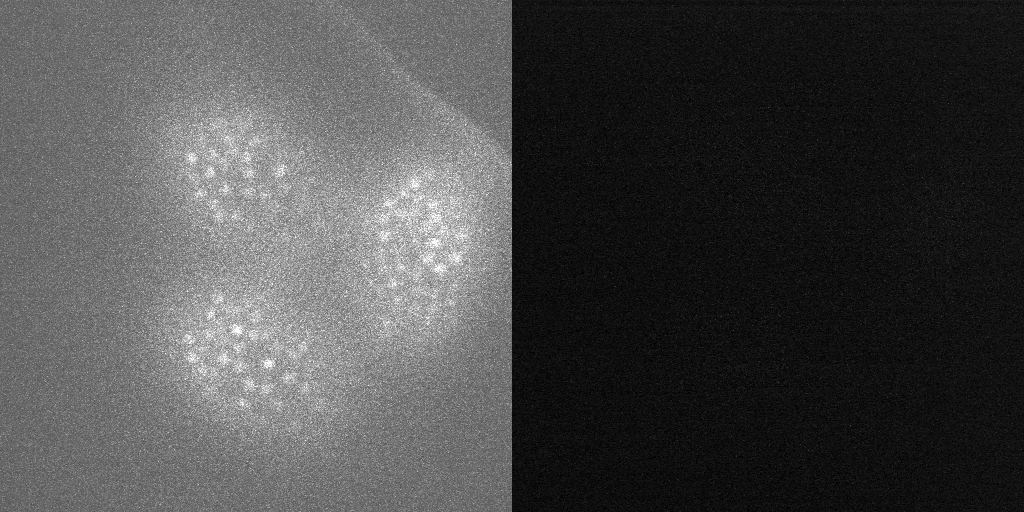

Supplement: Additional file 2 — Example image data, three images with ground truth segmentation. [file 1471-2105-15-217-S2.zip › ZD_RW10425_WT_20100412_2_s1_t161.TIF]
